# 1,3-Dibenzyl-2-(2-chloro­phen­yl)-4-methyl­imidazolidine

**DOI:** 10.1107/S1600536812047575

**Published:** 2012-11-24

**Authors:** Augusto Rivera, Lorena Cardenas, Jaime Ríos-Motta, Václav Eigner, Michal Dušek

**Affiliations:** a Universidad Nacional de Colombia, Sede Bogotá, Facultad de Ciencias, Departamento de Química, Cra 30 No. 45-03, Bogotá, Código Postal 111321, Colombia; b Department of Solid State Chemistry, Institute of Chemical Technology, Technická 5, 166 28 Prague, Czech Republic; c Institute of Physics AS CR, v.v.i., Na Slovance 2, 182 21 Prague 8, Czech Republic

## Abstract

In the title compound, C_24_H_25_ClN_2_, the methine, methyl­ene and methyl C atoms of the methyl-substituted imidazolidine ring are disordered over two sets of sites with a refined occupancy ratio of 0.834 (4):0.166 (4). Each disordered ring assumes an envelope conformation with an N atom as the flap. The pendant benzyl rings are oriented equatorially with respect to the imidazolidine ring. The chloro­phenyl ring is inclined to the mean plane of the four planar atoms of the major component of the imidazolidine ring by 76.27 (12)°. The dihedral angles between the chloro­phenyl ring and the two benzyl rings are 55.31 (9) and 57.50 (8)°; the dihedral angle between these latter rings is 71.59 (9)°. In the crystal, mol­ecules are linked by C—H⋯Cl inter­actions and a number of weak C—H⋯π inter­actions, involving all three aromatic rings, forming a three-dimensional structure.

## Related literature

For uses of imidazolidine-bridged bis­(phenol) derivatives in coordination chemistry, see: Xu *et al.* (2007 ▶). For related structures, see: Yang *et al.* (2009 ▶); Xia *et al.* (2007 ▶). For standard bond lengths, see: Allen *et al.* (1987 ▶). For ring conformations, see: Cremer & Pople (1975 ▶).
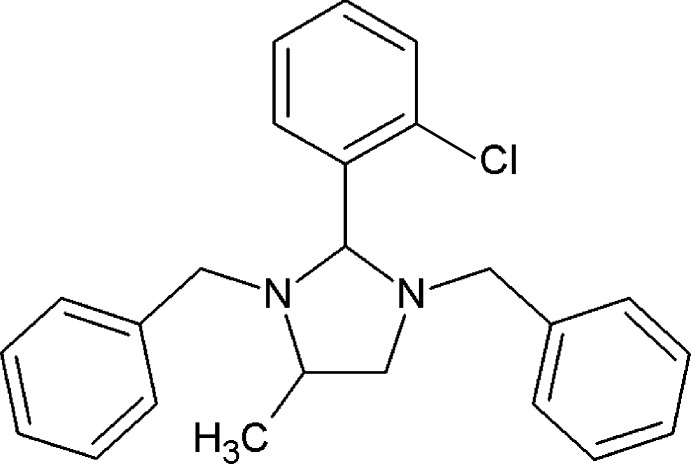


## Experimental

### 

#### Crystal data

C_24_H_25_ClN_2_
*M* *_r_* = 376.9
Monoclinic, 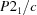

*a* = 7.1858 (1) Å
*b* = 9.8577 (2) Å
*c* = 29.3310 (5) Å
β = 96.8591 (15)°
*V* = 2062.80 (6) Å^3^
*Z* = 4
Cu *K*α radiation
μ = 1.70 mm^−1^
*T* = 120 K
0.44 × 0.32 × 0.21 mm

#### Data collection

Agilent Xcalibur (Atlas, Gemini ultra) diffractometer
Absorption correction: analytical (*CrysAlis PRO*; Agilent, 2010 ▶) *T*_min_ = 0.62, *T*_max_ = 0.751
39415 measured reflections
3665 independent reflections
3464 reflections with *I* > 3σ(*I*)
*R*_int_ = 0.029

#### Refinement

*R*[*F* ^2^ > 2σ(*F* ^2^)] = 0.043
*wR*(*F* ^2^) = 0.144
*S* = 2.91
3665 reflections
260 parameters
4 restraints
H-atom parameters constrained
Δρ_max_ = 0.24 e Å^−3^
Δρ_min_ = −0.35 e Å^−3^

### 

Data collection: *CrysAlis PRO* (Agilent, 2010 ▶); cell refinement: *CrysAlis PRO*; data reduction: *CrysAlis PRO*; program(s) used to solve structure: *SUPERFLIP* (Palatinus & Chapuis, 2007 ▶); program(s) used to refine structure: *JANA2006* (Petříček *et al.*, 2006 ▶); molecular graphics: *DIAMOND* (Brandenburg & Putz, 2005 ▶); software used to prepare material for publication: *JANA2006*.

## Supplementary Material

Crystal structure: contains datablock(s) global, I. DOI: 10.1107/S1600536812047575/su2527sup1.cif

Structure factors: contains datablock(s) I. DOI: 10.1107/S1600536812047575/su2527Isup2.hkl

Supplementary material file. DOI: 10.1107/S1600536812047575/su2527Isup3.cml

Additional supplementary materials:  crystallographic information; 3D view; checkCIF report

## Figures and Tables

**Table 1:** Hydrogen-bond geometry (Å, °) *Cg*1, *Cg*2 and *Cg*3 are the centroids of the C1–C6, C12–C17 and C18–C23 rings, respectively.

*D*—H⋯*A*	*D*—H	H⋯*A*	*D*⋯*A*	*D*—H⋯*A*
C9—H1C9⋯Cl24	0.96	2.58	3.1596 (16)	119
C21—H1*c*21⋯*Cg*2^i^	0.96	2.87	3.6324 (19)	137
C11—H2*c*11⋯*Cg*2^ii^	0.96	2.82	3.6192 (19)	142
C4—H1*c*4⋯*Cg*3^iii^	0.96	2.79	3.695 (2)	157
C26—H1*c*26⋯*Cg*1^iii^	0.96	2.91	3.688 (2)	139

Symmetry codes: (i) 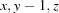; (ii) 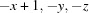; (iii) 
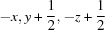
.

**Fig. 1.**
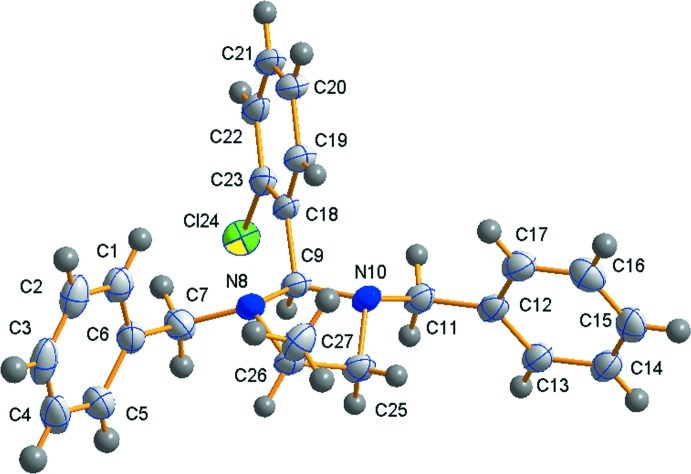
A view of the molecular structure of the title compound, showing the atom numbering. Displacement ellipsoids are drawn at the 50% probability level. Only the major component of the disordered methyl substitued imidazolidine ring is shown.

## supplementary crystallographic information

### Comment

As part of our studies on the synthesis of new imidazolidine derivatives we have prepared the title compound. It is an imidazolidine-bridged bis(phenol) which can serve as a useful precursor for the synthesis of lanthanide complexes of great potential application in homogeneous catalysis (Xu *et al.*, 2007), and herein we report on its crystal structure.

In the title compound, Fig. 1, the methyl substituted imidazolidine ring exhibits molecular disorder over two orientations, with a refined occupancy ratio of 0.834 (4):0.166 (4) for atoms (C25,C26,C27):(C25',C26',C27'). The bond lengths (Allen *et al.*, 1987) and angles are close to normal. In the imidazolidine ring, the bond lengths and angles are similar to those reported for closely related structures (Yang *et al.*, 2009; Xia *et al.*, 2007).

Each disordered component of the imidazolidine ring [N8/C9/N10/C25(25')/C26(26')] adopts an envelope conformation on N10 (major component) and N8 (minor component), respectively, with puckering parameters of Q2 = 0.427 (2) Å and φ2 = 252.5 (3)° for the major component, and Q2 = 0.555 (6) Å and φ2 = 178.7 (8)° for the minor component [Cremer & Pople, 1975].

The chlorophenyl ring attached to C9 (C18—C23) is inclined to the mean plane of the four planar atoms of the major component of the imidazolidine ring by 76.27 (12) °. The dihedral angles between the chlorophenyl ring (C18–C23) and the two benzyl rings (C1-C6) and (C12-C17) are 55.31 (9) and 57.50 (8)°, respectively. The pendant phenyl rings of the benzyl groups are oriented equatorially to the imidazolidine ring. The dihedral angle between these rings is 71.59 (9)°.

In the crystal, molecules are linked by C-H···Cl interactions and a number of weak C—H···π interactions, involving all three aromatic rings (Table 1), forming a three-dimensional structure.

### Experimental

A toluene solution of *N*^1^,*N*^2^-dibenzylpropane-1,2-diamine was refluxed for 8 h with 4*-*chlorobenzaldehyde in a molar ratio of 1.1:1.0. The mixture was evaporated on a rotary evaporator. The residue was cooled, and the precipitate was filtered off. It was then washed with cold ethanol, dried in air, and recrystallized from ethanol [Yield 81%; M.p. 352-353 K].

### Refinement

H atoms present in the structural model were discernible in difference Fourier maps and could be refined to reasonable geometry. According to common practice H atoms bonded to C were kept in ideal positions with C–H = 0.96 Å and U_iso_(H) = 1.2U_eq_(C,N). The methine, methylene and methyl groups of the methyl substituted imidazolidine ring were found to be disordered with a refined occupancy ratio of 0.834 (4):0.166 (4). The disordered part of the molecule was refined with bond distances of both fractions kept at the same values. The H atoms of the minor fraction could also be found in difference Fourier maps as faint maxima, however, their addition had negligible impact on R values and GOF. Moreover, it was found that the refined geometry of the minor fraction C atoms is not sufficiently correct for derivation of proper H atom positions, as indicated by too close positions between H2C25' and H2C7 (1.81Å). However, these H atoms were retained in the final refined structural model.

### Crystal data

**Table:**

C_24_H_25_ClN_2_	*Z* = 4
*M**_r_* = 376.9	*F*(000) = 800
Monoclinic, *P*2_1_/*c*	*D*_x_ = 1.213 Mg m^−^^3^
Hall symbol: -P 2ycb	Cu *K*α radiation, λ = 1.5418 Å
*a* = 7.1858 (1) Å	Cell parameters from 20478 reflections
*b* = 9.8577 (2) Å	θ = 3.0–67.0°
*c* = 29.3310 (5) Å	µ = 1.70 mm^−^^1^
β = 96.8591 (15)°	*T* = 120 K
*V* = 2062.80 (6) Å^3^	0.44 × 0.32 × 0.21 mm

### Data collection

**Table:**

Agilent Xcalibur (Atlas, Gemini ultra) diffractometer	3665 independent reflections
Radiation source: Enhance Ultra (Cu) X-ray Source	3464 reflections with *I* > 3σ(*I*)
Mirror monochromator	*R*_int_ = 0.029
Detector resolution: 10.3784 pixels mm^-1^	θ_max_ = 67.1°, θ_min_ = 3.0°
ω scans	*h* = −8→8
Absorption correction: analytical (*CrysAlis PRO*; Agilent, 2010)	*k* = −11→11
*T*_min_ = 0.62, *T*_max_ = 0.751	*l* = −34→34
39415 measured reflections	

### Refinement

**Table:**

Refinement on *F*^2^	153 constraints
*R*[*F*^2^ > 2σ(*F*^2^)] = 0.043	H-atom parameters constrained
*wR*(*F*^2^) = 0.144	Weighting scheme based on measured s.u.'s *w* = 1/(σ^2^(*I*) + 0.0016*I*^2^)
*S* = 2.91	(Δ/σ)_max_ = 0.047
3665 reflections	Δρ_max_ = 0.24 e Å^−^^3^
260 parameters	Δρ_min_ = −0.35 e Å^−^^3^
4 restraints	

### Fractional atomic coordinates and isotropic or equivalent isotropic displacement parameters (Å^2^)

**Table:**

	*x*	*y*	*z*	*U*_iso_*/*U*_eq_	Occ. (<1)
Cl24	−0.02857 (6)	−0.36115 (5)	0.065592 (15)	0.04745 (17)	
N8	0.1847 (2)	−0.10600 (13)	0.17251 (5)	0.0361 (4)	
N10	0.3151 (2)	−0.02081 (12)	0.11112 (5)	0.0344 (4)	
C16	0.7452 (3)	0.2032 (2)	0.05130 (6)	0.0477 (6)	
C20	0.5614 (3)	−0.41315 (17)	0.13435 (6)	0.0424 (6)	
C2	0.1697 (3)	−0.20165 (19)	0.31553 (7)	0.0497 (6)	
C19	0.4692 (2)	−0.29057 (17)	0.13876 (6)	0.0391 (5)	
C15	0.6776 (3)	0.30121 (19)	0.01963 (6)	0.0452 (6)	
C17	0.6261 (3)	0.10445 (18)	0.06521 (6)	0.0448 (6)	
C21	0.4719 (3)	−0.51645 (17)	0.10812 (6)	0.0427 (6)	
C1	0.1636 (3)	−0.21435 (18)	0.26802 (6)	0.0432 (6)	
C3	0.0314 (3)	−0.13050 (19)	0.33373 (7)	0.0553 (7)	
C23	0.2014 (2)	−0.37547 (16)	0.09207 (6)	0.0349 (5)	
C22	0.2929 (3)	−0.49840 (16)	0.08697 (6)	0.0399 (5)	
C18	0.2879 (2)	−0.26912 (15)	0.11737 (5)	0.0324 (5)	
C11	0.3064 (3)	−0.00554 (16)	0.06139 (6)	0.0390 (5)	
C6	0.0205 (3)	−0.15668 (17)	0.23914 (6)	0.0406 (6)	
C12	0.4379 (3)	0.10288 (16)	0.04778 (5)	0.0362 (5)	
C9	0.1973 (2)	−0.13226 (15)	0.12385 (6)	0.0340 (5)	
C14	0.4925 (3)	0.29944 (18)	0.00181 (6)	0.0446 (6)	
C13	0.3724 (3)	0.20095 (16)	0.01582 (6)	0.0398 (5)	
C7	0.0183 (2)	−0.1681 (2)	0.18783 (6)	0.0434 (6)	
C25	0.2377 (4)	0.0968 (2)	0.13284 (8)	0.0382 (6)	0.834 (4)
C26'	0.2578 (14)	0.0937 (8)	0.1408 (4)	0.0382 (6)	0.166 (4)
C4	−0.1125 (3)	−0.0734 (2)	0.30561 (8)	0.0593 (8)	
C5	−0.1196 (3)	−0.0859 (2)	0.25804 (7)	0.0510 (7)	
C27	0.3731 (3)	0.0772 (2)	0.21620 (8)	0.0475 (7)	0.834 (4)
C26	0.2097 (3)	0.04486 (18)	0.18044 (7)	0.0349 (7)	0.834 (4)
C27'	0.4362 (15)	0.1242 (11)	0.1719 (4)	0.047 (4)	0.166 (4)
C25'	0.1081 (17)	0.0315 (6)	0.1670 (4)	0.0349 (7)	0.166 (4)
H1c16	0.875012	0.203631	0.063679	0.0573*	
H1c20	0.686283	−0.426164	0.149381	0.0509*	
H1c2	0.269944	−0.242418	0.335449	0.0596*	
H1c19	0.531872	−0.21934	0.156898	0.047*	
H1c15	0.760026	0.369776	0.01026	0.0543*	
H1c17	0.674392	0.036817	0.087035	0.0538*	
H1c21	0.535525	−0.600857	0.104771	0.0513*	
H1c1	0.260406	−0.263872	0.255352	0.0518*	
H1c3	0.036117	−0.120936	0.366424	0.0664*	
H1c22	0.231051	−0.569829	0.068775	0.0479*	
H1c11	0.180416	0.015881	0.048777	0.0468*	
H2c11	0.336042	−0.090551	0.04801	0.0468*	
H1c9	0.077614	−0.135976	0.105426	0.0408*	
H1c14	0.445321	0.366312	−0.020417	0.0535*	
H1c13	0.242801	0.200852	0.003238	0.0478*	
H1c7	0.01236	−0.26198	0.179091	0.0521*	
H2c7	−0.092009	−0.124555	0.172859	0.0521*	
H1c4	−0.209051	−0.024474	0.318574	0.0711*	
H1c5	−0.221036	−0.045807	0.238336	0.0612*	
H1c25	0.328399	0.168788	0.135627	0.0458*	0.834 (4)
H2c25	0.118473	0.120137	0.116385	0.0458*	0.834 (4)
H1c27	0.487331	0.046259	0.205703	0.057*	0.834 (4)
H2c27	0.379789	0.173462	0.221124	0.057*	0.834 (4)
H3c27	0.356064	0.032474	0.244474	0.057*	0.834 (4)
H1c26	0.105513	0.086723	0.192608	0.0419*	0.834 (4)
H1c26'	0.209453	0.174743	0.125584	0.0458*	0.166 (4)
H1c25'	0.111895	0.073924	0.196512	0.0419*	0.166 (4)
H2c25'	−0.009138	0.028882	0.147483	0.0419*	0.166 (4)
H1c27'	0.497636	0.040672	0.181514	0.0568*	0.166 (4)
H2c27'	0.517824	0.178023	0.155616	0.0568*	0.166 (4)
H3c27'	0.406452	0.173148	0.198402	0.0568*	0.166 (4)

### Atomic displacement parameters (Å^2^)

**Table:**

	*U*^11^	*U*^22^	*U*^33^	*U*^12^	*U*^13^	*U*^23^
Cl24	0.0489 (3)	0.0456 (3)	0.0467 (3)	0.00039 (17)	0.0010 (2)	−0.00135 (17)
N8	0.0483 (8)	0.0258 (7)	0.0366 (7)	0.0073 (5)	0.0153 (6)	0.0017 (5)
N10	0.0490 (8)	0.0239 (6)	0.0314 (7)	0.0070 (5)	0.0101 (6)	0.0025 (5)
C16	0.0496 (10)	0.0535 (11)	0.0411 (10)	0.0026 (8)	0.0098 (8)	−0.0068 (8)
C20	0.0465 (10)	0.0350 (9)	0.0471 (10)	0.0140 (7)	0.0113 (8)	0.0070 (7)
C2	0.0659 (12)	0.0420 (10)	0.0414 (10)	−0.0193 (9)	0.0072 (9)	0.0011 (8)
C19	0.0453 (9)	0.0294 (8)	0.0434 (9)	0.0075 (7)	0.0082 (8)	0.0015 (7)
C15	0.0613 (11)	0.0405 (9)	0.0372 (9)	−0.0035 (8)	0.0201 (8)	−0.0063 (7)
C17	0.0572 (11)	0.0400 (9)	0.0379 (9)	0.0119 (8)	0.0081 (8)	0.0038 (7)
C21	0.0600 (11)	0.0303 (8)	0.0416 (9)	0.0145 (7)	0.0213 (9)	0.0054 (7)
C1	0.0512 (10)	0.0357 (9)	0.0437 (10)	−0.0076 (7)	0.0101 (8)	−0.0007 (7)
C3	0.0879 (16)	0.0390 (10)	0.0423 (11)	−0.0265 (10)	0.0210 (11)	−0.0051 (8)
C23	0.0434 (9)	0.0314 (8)	0.0315 (8)	0.0037 (6)	0.0114 (7)	0.0047 (6)
C22	0.0612 (11)	0.0268 (8)	0.0346 (9)	0.0023 (7)	0.0178 (8)	0.0006 (6)
C18	0.0436 (9)	0.0244 (7)	0.0310 (7)	0.0061 (6)	0.0119 (7)	0.0032 (6)
C11	0.0563 (10)	0.0289 (8)	0.0328 (8)	0.0049 (7)	0.0090 (8)	0.0008 (6)
C6	0.0492 (10)	0.0338 (9)	0.0416 (10)	−0.0023 (7)	0.0175 (8)	0.0007 (7)
C12	0.0541 (10)	0.0270 (8)	0.0290 (8)	0.0070 (7)	0.0111 (7)	−0.0011 (6)
C9	0.0408 (9)	0.0265 (8)	0.0351 (9)	0.0083 (6)	0.0069 (7)	0.0022 (6)
C14	0.0663 (12)	0.0326 (9)	0.0368 (9)	0.0053 (8)	0.0142 (8)	0.0040 (7)
C13	0.0532 (10)	0.0331 (8)	0.0342 (8)	0.0067 (7)	0.0093 (8)	0.0025 (7)
C7	0.0403 (9)	0.0503 (10)	0.0409 (10)	0.0051 (7)	0.0099 (8)	0.0029 (8)
C25	0.0524 (11)	0.0271 (8)	0.0357 (11)	0.0063 (7)	0.0082 (9)	−0.0031 (8)
C26'	0.0524 (11)	0.0271 (8)	0.0357 (11)	0.0063 (7)	0.0082 (9)	−0.0031 (8)
C4	0.0835 (15)	0.0404 (10)	0.0624 (13)	−0.0097 (10)	0.0439 (12)	−0.0095 (9)
C5	0.0574 (12)	0.0454 (10)	0.0544 (11)	0.0045 (8)	0.0241 (10)	0.0034 (9)
C27	0.0632 (14)	0.0351 (11)	0.0427 (12)	−0.0055 (10)	0.0004 (10)	0.0021 (9)
C26	0.0446 (14)	0.0231 (8)	0.0384 (11)	0.0061 (8)	0.0109 (10)	−0.0018 (7)
C27'	0.044 (6)	0.042 (6)	0.056 (7)	−0.006 (4)	0.007 (5)	−0.018 (5)
C25'	0.0446 (14)	0.0231 (8)	0.0384 (11)	0.0061 (8)	0.0109 (10)	−0.0018 (7)

### Geometric parameters (Å, º)

**Table:**

N8—C9	1.464 (2)	C18—C9	1.520 (2)
N8—C7	1.461 (2)	C11—C12	1.511 (2)
N8—C26	1.513 (2)	C11—H1c11	0.96
N8—C25'	1.464 (7)	C11—H2c11	0.96
N10—C11	1.460 (2)	C6—C7	1.507 (3)
N10—C9	1.462 (2)	C6—C5	1.393 (3)
N10—C25	1.464 (3)	C12—C13	1.389 (2)
N10—C26'	1.512 (10)	C9—H1c9	0.96
C16—C15	1.387 (3)	C14—C13	1.393 (3)
C16—C17	1.389 (3)	C14—H1c14	0.96
C16—H1c16	0.96	C13—H1c13	0.96
C20—C19	1.391 (2)	C7—H1c7	0.96
C20—C21	1.387 (2)	C7—H2c7	0.96
C20—H1c20	0.96	C25—C26	1.523 (3)
C2—C1	1.395 (3)	C25—H1c25	0.96
C2—C3	1.375 (3)	C25—H2c25	0.96
C2—H1c2	0.96	C26'—C27'	1.512 (14)
C19—C18	1.393 (2)	C26'—C25'	1.523 (16)
C19—H1c19	0.96	C26'—H1c26'	0.96
C15—C14	1.370 (3)	C4—C5	1.395 (3)
C15—H1c15	0.96	C4—H1c4	0.96
C17—C12	1.388 (3)	C5—H1c5	0.96
C17—H1c17	0.96	C27—C26	1.512 (3)
C21—C22	1.371 (3)	C27—H1c27	0.96
C21—H1c21	0.96	C27—H2c27	0.96
C1—C6	1.375 (2)	C27—H3c27	0.96
C1—H1c1	0.96	C26—H1c26	0.96
C3—C4	1.365 (3)	C27'—H1c27'	0.96
C3—H1c3	0.96	C27'—H2c27'	0.96
C23—C22	1.395 (2)	C27'—H3c27'	0.96
C23—C18	1.388 (2)	C25'—H1c25'	0.96
C22—H1c22	0.96	C25'—H2c25'	0.96
			
C9—N8—C7	111.94 (13)	N8—C9—H1c9	113.6
C9—N8—C26	107.69 (13)	N10—C9—C18	111.41 (14)
C9—N8—C25'	97.0 (5)	N10—C9—H1c9	113.07
C7—N8—C26	116.80 (15)	C18—C9—H1c9	105.31
C7—N8—C25'	96.4 (5)	C15—C14—C13	120.25 (16)
C11—N10—C9	112.07 (12)	C15—C14—H1c14	119.88
C11—N10—C25	112.33 (14)	C13—C14—H1c14	119.88
C11—N10—C26'	121.1 (4)	C12—C13—C14	120.82 (17)
C9—N10—C25	102.85 (15)	C12—C13—H1c13	119.59
C9—N10—C26'	102.0 (4)	C14—C13—H1c13	119.59
C15—C16—C17	120.43 (18)	N8—C7—C6	111.35 (14)
C15—C16—H1c16	119.79	N8—C7—H1c7	109.47
C17—C16—H1c16	119.78	N8—C7—H2c7	109.47
C19—C20—C21	119.66 (16)	C6—C7—H1c7	109.47
C19—C20—H1c20	120.17	C6—C7—H2c7	109.47
C21—C20—H1c20	120.17	H1c7—C7—H2c7	107.53
C1—C2—C3	119.75 (18)	N10—C25—C26	103.37 (16)
C1—C2—H1c2	120.13	N10—C25—H1c25	109.47
C3—C2—H1c2	120.13	N10—C25—H2c25	109.47
C20—C19—C18	121.33 (15)	C26—C25—H1c25	109.47
C20—C19—H1c19	119.33	C26—C25—H2c25	109.47
C18—C19—H1c19	119.33	H1c25—C25—H2c25	114.95
C16—C15—C14	119.51 (18)	N10—C26'—C27'	103.1 (7)
C16—C15—H1c15	120.24	N10—C26'—C25'	104.3 (6)
C14—C15—H1c15	120.25	N10—C26'—H1c26'	117.48
C16—C17—C12	120.46 (16)	C27'—C26'—C25'	112.0 (9)
C16—C17—H1c17	119.77	C27'—C26'—H1c26'	110.43
C12—C17—H1c17	119.77	C25'—C26'—H1c26'	109.35
C20—C21—C22	120.22 (16)	C3—C4—C5	120.1 (2)
C20—C21—H1c21	119.89	C3—C4—H1c4	119.92
C22—C21—H1c21	119.89	C5—C4—H1c4	119.93
C2—C1—C6	120.67 (18)	C6—C5—C4	120.03 (19)
C2—C1—H1c1	119.66	C6—C5—H1c5	119.98
C6—C1—H1c1	119.66	C4—C5—H1c5	119.98
C2—C3—C4	120.4 (2)	C26—C27—H1c27	109.47
C2—C3—H1c3	119.8	C26—C27—H2c27	109.47
C4—C3—H1c3	119.8	C26—C27—H3c27	109.47
C22—C23—C18	121.77 (15)	H1c27—C27—H2c27	109.47
C21—C22—C23	119.53 (15)	H1c27—C27—H3c27	109.47
C21—C22—H1c22	120.23	H2c27—C27—H3c27	109.47
C23—C22—H1c22	120.24	N8—C26—C25	102.47 (15)
C19—C18—C23	117.47 (14)	N8—C26—C27	112.46 (16)
C19—C18—C9	118.05 (13)	N8—C26—H1c26	113.36
C23—C18—C9	124.48 (14)	C25—C26—C27	112.82 (19)
N10—C11—C12	112.54 (13)	C25—C26—H1c26	113
N10—C11—H1c11	109.47	C27—C26—H1c26	103.12
N10—C11—H2c11	109.47	C26'—C27'—H1c27'	109.47
C12—C11—H1c11	109.47	C26'—C27'—H2c27'	109.47
C12—C11—H2c11	109.47	C26'—C27'—H3c27'	109.47
H1c11—C11—H2c11	106.21	H1c27'—C27'—H2c27'	109.47
C1—C6—C7	120.16 (17)	H1c27'—C27'—H3c27'	109.47
C1—C6—C5	119.00 (18)	H2c27'—C27'—H3c27'	109.47
C7—C6—C5	120.83 (16)	N8—C25'—C26'	98.6 (8)
C17—C12—C11	121.54 (15)	N8—C25'—H1c25'	109.47
C17—C12—C13	118.53 (16)	N8—C25'—H2c25'	109.47
C11—C12—C13	119.89 (15)	C26'—C25'—H1c25'	109.47
N8—C9—N10	102.77 (12)	C26'—C25'—H2c25'	109.47
N8—C9—C18	110.86 (12)	H1c25'—C25'—H2c25'	118.51

### Hydrogen-bond geometry (Å, º)

Cg1, Cg2 and Cg3 are the centroids of the C1–C6, C12–C17 and C18–C23 rings, respectively.

**Table:**

*D*—H···*A*	*D*—H	H···*A*	*D*···*A*	*D*—H···*A*
C9—H1C9···Cl24	0.96	2.58	3.1596 (16)	119
C21—H1*c*21···*Cg*2^i^	0.96	2.87	3.6324 (19)	137
C11—H2*c*11···*Cg*2^ii^	0.96	2.82	3.6192 (19)	142
C4—H1*c*4···*Cg*3^iii^	0.96	2.79	3.695 (2)	157
C26—H1*c*26···*Cg*1^iii^	0.96	2.91	3.688 (2)	139

Symmetry codes: (i) *x*, *y*−1, *z*; (ii) −*x*+1, −*y*, −*z*; (iii) −*x*, *y*+1/2, −*z*+1/2.
